# Purification and Structural Characterization of Aggregation-Prone Human TDP-43 Involved in Neurodegenerative Diseases

**DOI:** 10.1016/j.isci.2020.101159

**Published:** 2020-05-15

**Authors:** Gareth S.A. Wright, Tatiana F. Watanabe, Kangsa Amporndanai, Steven S. Plotkin, Neil R. Cashman, Svetlana V. Antonyuk, S. Samar Hasnain

**Affiliations:** 1 Molecular Biophysics Group, Department of Biochemistry & Systems Biology, Institute of Systems, Molecular and Integrative Biology, Faculty of Health and Life Sciences, Liverpool L69 7ZB, UK; 2 Department of Physics & Astronomy, The University of British Columbia, Vancouver, BC, Canada; 3 Djavad Mowafaghian Centre for Brain Health, University of British Columbia, Vancouver, BC V6T 2B5, Canada

**Keywords:** Molecular Neuroscience, Structural Biology, Protein Folding

## Abstract

Mislocalization, cleavage, and aggregation of the human protein TDP-43 is found in many neurodegenerative diseases. As is the case with many other proteins that are completely or partially structurally disordered, production of full-length recombinant TDP-43 in the quantities necessary for structural characterization has proved difficult. We show that the full-length TDP-43 protein and two truncated N-terminal constructs 1-270 and 1-263 can be heterologously expressed in *E. coli*. Full-length TDP-43 could be prevented from aggregation during purification using a detergent. Crystals grown from an N-terminal construct (1-270) revealed only the N-terminal domain (residues 1-80) with molecules arranged as parallel spirals with neighboring molecules arranged in head-to-tail fashion. To obtain detergent-free, full-length TDP-43 we mutated all six tryptophan residues to alanine. This provided sufficient soluble protein to collect small-angle X-ray scattering data. Refining relative positions of individual domains and intrinsically disordered regions against this data yielded a model of full-length TDP-43.

## Highlights

• Purification of full-length TDP-43 with all tryptophan residues mutated to alanine
• Crystallographic structure determination of the TDP-43 N-terminal domain
• Small-angle X-ray scattering analysis of full-length TDP-43
• Potential structural model of full-length TDP-43

## Graphical Abstract

**Figure:**
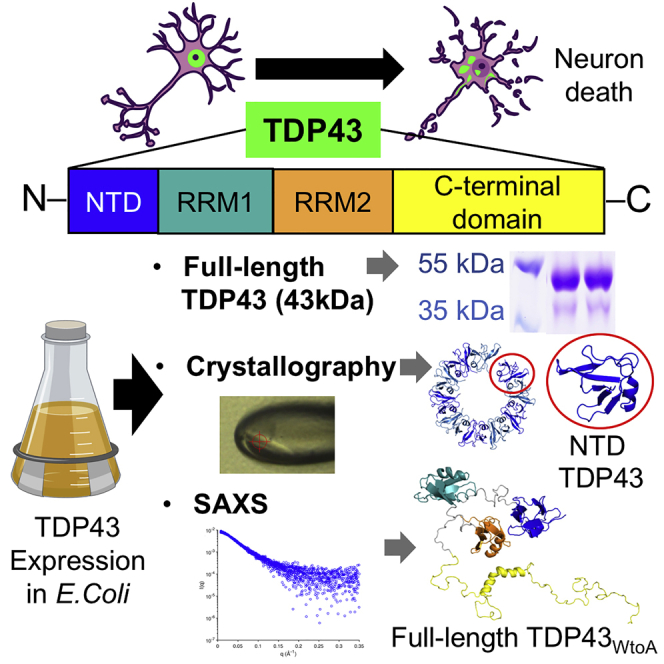

## Introduction

The deposition of intracellular TDP-43 inclusions is the hallmark of TDP-43 pathology. Initially observed in neural tissues from individuals with frontotemporal lobar degeneration (FTLD) and amyotrophic lateral sclerosis (ALS) (Neumann et al., 2006), TDP-43 pathology is now associated with many neurodegenerative diseases. These include, but are by no means limited to, Alzheimer disease (Amador-Ortiz et al., 2007), Parkinson disease (Nakashima-Yasuda et al., 2007), hippocampal sclerosis (Amador-Ortiz et al., 2007), and chronic traumatic encephalopathy (McKee et al., 2010). Mixed pathology is common with these diseases, and TDP-43 pathology can be found not only alongside Lewy bodies (Nakashima-Yasuda et al., 2007), amyloid-β plaques, and tau tangles (Amador-Ortiz et al., 2007) in cases of neurodegenerative disease but also in clinically normal aged individuals (Wennberg et al., 2019).

TDP-43 protein has several functions, and its modular structure facilitates this multitasking. Two centrally located RNA recognition motifs (RRM) strongly bind UG-rich RNA (Lukavsky et al., 2013) or TG-rich DNA (Austin et al., 2014) directing TDP-43 to pre-mRNAs and intronic sites (Tollervey et al., 2011). Through these protein-nucleic acid interactions TDP-43 facilitates RNA transport (Fallini et al., 2012) and directly effects splicing of a multitude of RNAs including those coding for many ALS-associated heterogeneous nuclear ribonucleoprotein particles (Deshaies et al., 2018) and TDP-43 itself (Ayala et al., 2011). A low-complexity domain, situated C terminal to the RRM domains, is involved in stress granule formation following cellular stress (Colombrita et al., 2009). This domain undergoes liquid-liquid phase transitions and complexes with other TDP-43 molecules (Li et al., 2018a) or other intrinsically disordered proteins (McDonald et al., 2011), whereas RRM domains trap mRNAs to assist selective translation during and following stress. Many single amino acid substitutions within the C-terminal domain are known to cause ALS and FTLD, indicating that aberrant stress granule dynamics may lie at the heart of TDP-43 proteotoxicity (Wolozin, 2012). At the N terminus, a ubiquitin or dix-like domain (Mompeán et al., 2016, Qin et al., 2014) provides a polymerization surface that enables formation of dimer and higher-order oligomers predominantly found in the cell nucleus (Afroz et al., 2017).

In addition to the domains described earlier, TDP-43 also contains nuclear export and import sequences that flank the RRM domains. In the full-length form, this enables shuttling of the protein between the nucleus and cytoplasm (Ayala et al., 2008). Observation of TDP-43 inclusions in FTLD and ALS brain tissues has shown that it is fragmented (Neumann et al., 2006) with a 25-kDa cleavage product being the most common but 15- and 35-kDa forms are also seen. The common element present in these cleavage products is the low-complexity C-terminal domain. In this state, the nuclear localization signal is lost and the protein remains cytoplasmic and aggregates through the low-complexity C-terminal domain. The normal functions and misfunction of TDP-43 are therefore predicated by its propensity to oligomerize and aggregate. This property makes *in vitro* characterization difficult, particularly using structural techniques that require monodisperse samples at relatively high concentrations. The modularity of TDP-43 means that individual domains can be produced recombinantly to shed light on their organization and structure-property relationship. This approach has been well utilized to gain insight on dimerization (Afroz et al., 2017), RNA binding (Lukavsky et al., 2013), and thermal stability (Austin et al., 2014, Chiang et al., 2016). However, to go beyond individual domains and gain a holistic understanding of TDP-43 structure we tested several strategies to produce pure, full-length TDP-43. Using sarkosyl detergent and mutagenically removing tryptophan resides (TDP-43_WtoA_), we were able prevent recombinant TDP-43 aggregation during protein preparation in each of these cases. We produced sufficient quantities of detergent-free TDP-43_WtoA_ for small-angle X-ray scattering (SAXS) analysis allowing us to create a model of full-length TDP-43.

## Results

### Recombinant Expression and Purification of Full-Length and C-Terminal Truncated TDP-43

**Figure 1:**
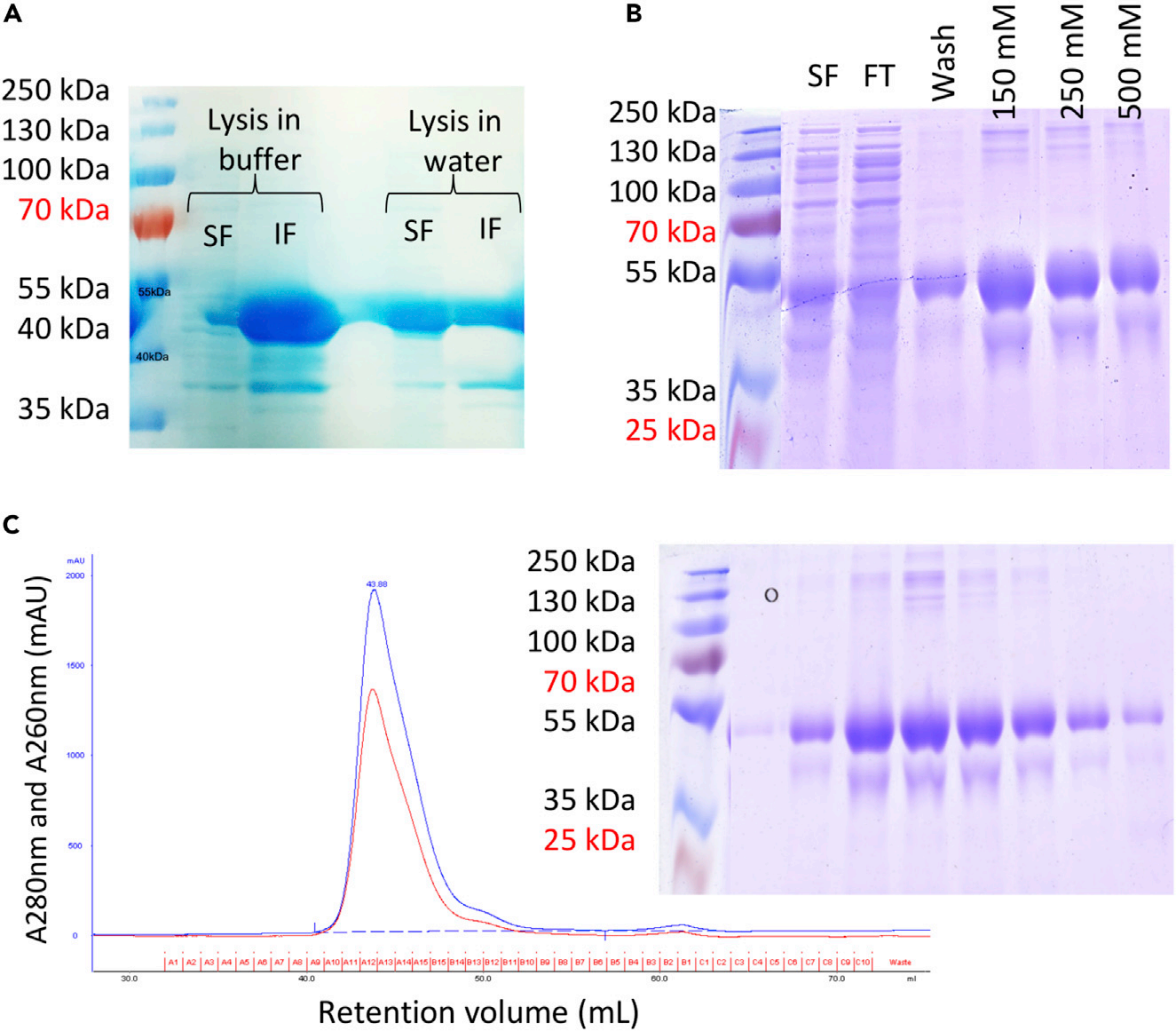
Purification of Full-Length Wild-Type TDP-43 Using 0.2% Sarkosyl (A) Cell lysis in 50 mM sodium phosphate pH 8.0, 300 mM sodium chloride, 5 mM imidazole, 5 mM dithiothreitol, complete protease inhibitor cocktail, 1 mM phenylmethylsulphonyl fluoride, 50 μg/mL lysozyme, and pure water. SF, soluble fraction; IF, insoluble fraction. (B) Immobilized metal ion chromatography. SF, soluble fraction; FT, flowthrough; wash fraction (75 mM imidazole), elution with increasing imidazole concentration. (C) Size exclusion chromatography (SEC) and SDS-PAGE of SEC fractions containing pure wild-type TDP-43.

On heterologous expression of full-length wild-type TDP-43 in *E. coli* followed by cell lysis and centrifugation, we found the protein in the insoluble fraction as has previously been described (Furukawa et al., 2011). However, low-temperature expression followed by cell lysis in pure water yielded segregation of the protein roughly equally between soluble and insoluble fractions but made affinity purification problematic. This is possibly an indication that recombinant TDP-43 is not localized to inclusion bodies when expressed in bacteria. To solve this problem, 0.2% sodium lauroyl sarcosinate (sarkosyl) was added to the cell lysate and used in all purification buffers. This yielded 5.3 mg of pure, soluble protein for a litre of culture (Figure 1). This detergent-solubilized protein is stable without visible aggregation after a week at 4°C or a freeze/thaw cycle.

**Figure 2:**
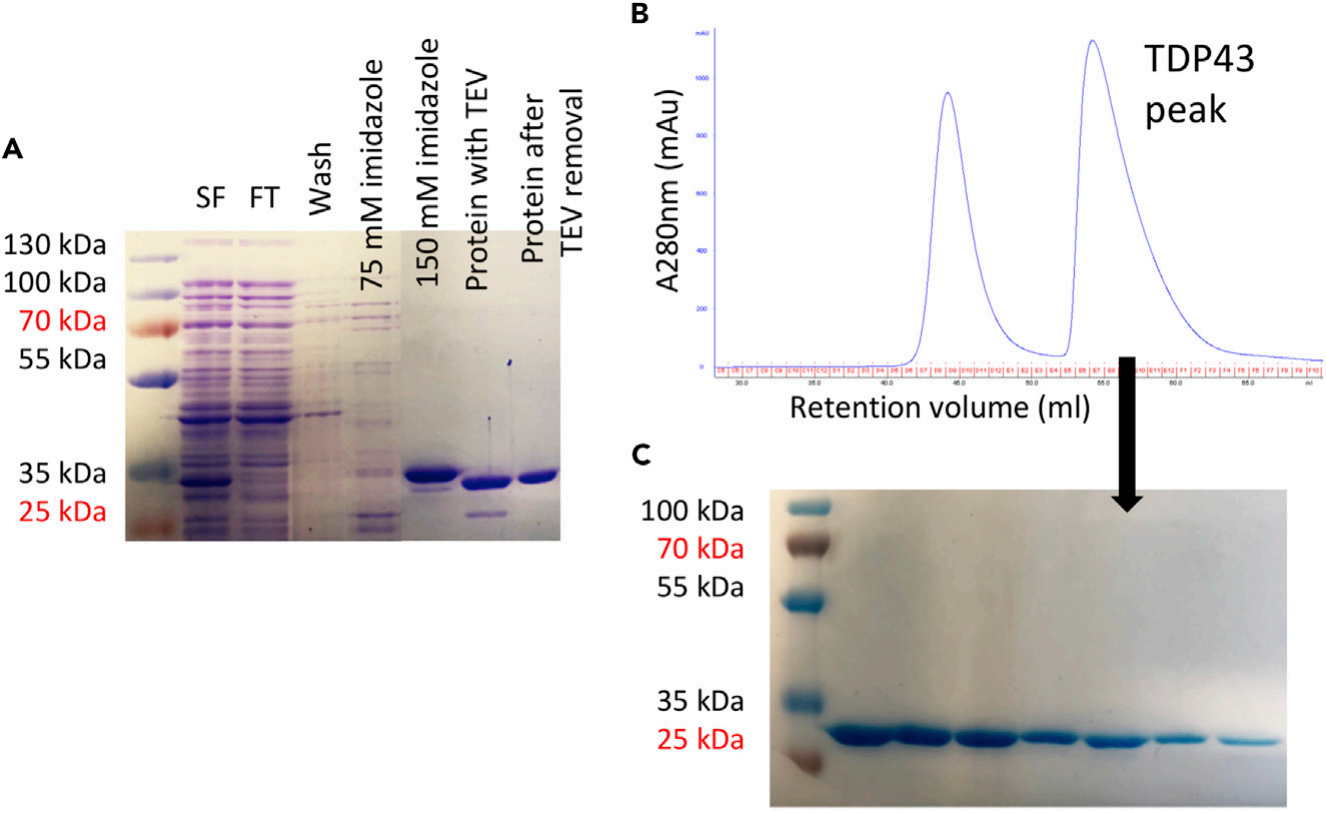
Purification of Wild-Type TDP-43 Residues 1-270 (A) Nickel-nitriloacetic acid (NiNTA)-immobilized metal ion chromatography (IMAC). SF, soluble fraction; FT, flowthrough; wash (5 mM imidazole); 75 mM/150 mM elution imidazole concentration. (B) Size exclusion chromatography. (C) Fractions from (B) containing pure wild-type TDP-43 residues 1-270.

Several constructs of C-terminal truncated TDP-43 (N-terminal 1-263, 1-270, 1-290, 1-320, and several single point mutations of 1-270 fragment including A90V, D169G, K263E, and N267S) were prepared and evaluated. The N-terminal 1-270 wild-type construct provided stable purified protein (Figure 2) and was pursued for further investigations. This was used for crystallization experiments from which structure of N-terminal domain (NTD) (1-80) was obtained.

**Figure 3:**
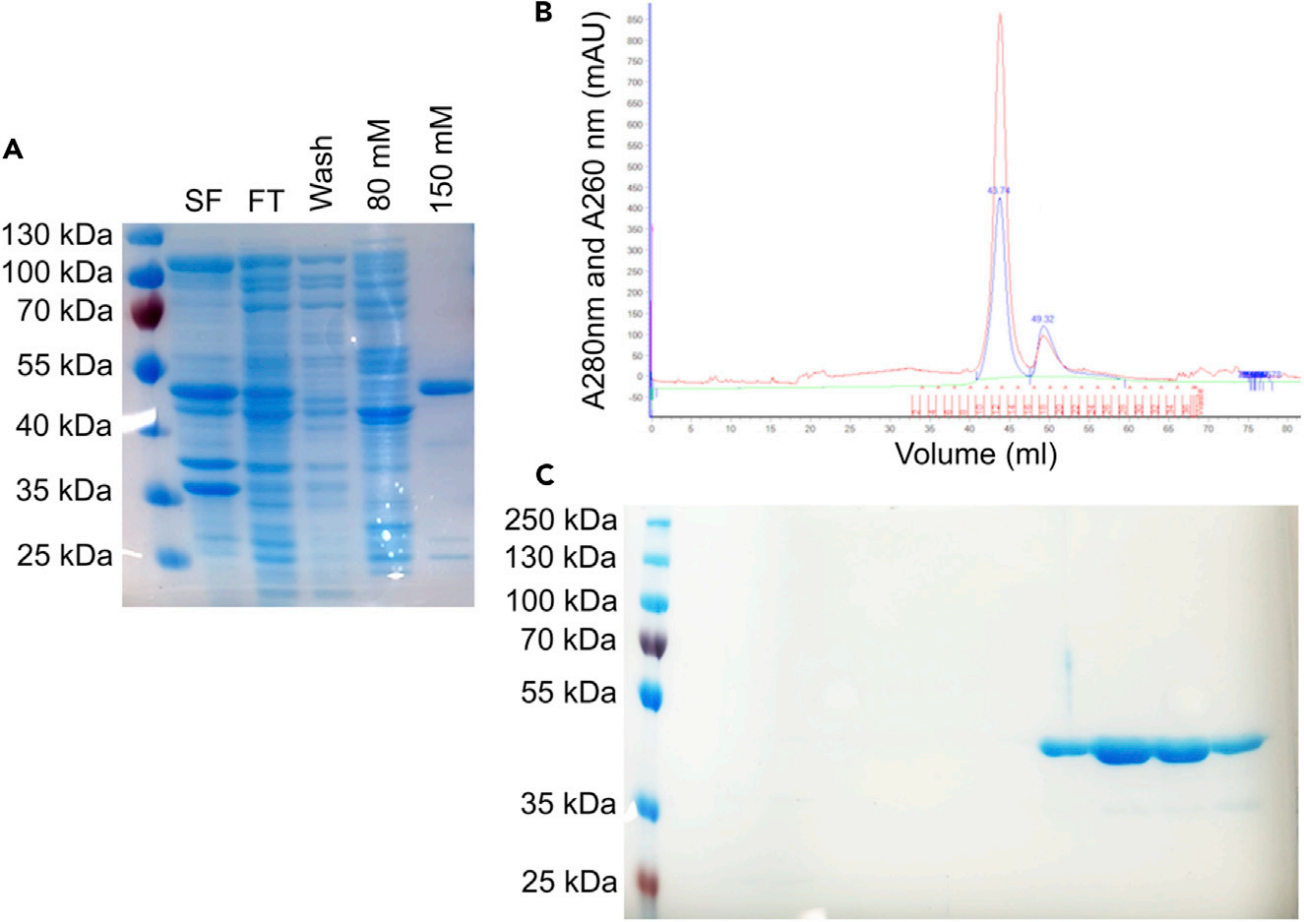
Purification of Full-Length TDP-43_WtoA_ (A) NiNTA IMAC. SF, soluble fraction; FT, flowthrough; wash (5 mM imidazole); 80 mM/150 mM elution imidazole concentration. (B) Size exclusion chromatography (SEC). (C) SEC fractions containing pure TDP-43_WtoA_.

Addition of soluble fusion domains is a common technique used to enable production of recombinant proteins. Wang et al. (2018) added a maltose-binding protein fusion to TDP-43 directly C terminal to the low-complexity domain where it effectively increased solubility. To obtain detergent-free TDP-43 without recourse to fusions proteins, and with the knowledge that several TDP-43 tryptophan residues are involved in folding (Prakash et al., 2018) and phase transitioning (Li et al., 2018a, Li et al., 2018b), together with the role of aromatic residues in low-complexity aromatic-rich kinked segment formation (Hughes et al., 2018), we created a construct where all tryptophan residues in the TDP-43 primary sequence are replaced with alanine (TDP-43_WtoA_). Cell lysis and protein purification with standard immobilized metal ion chromatography buffers without any detergent facilitated production of full-length TDP-43_WtoA_ that was stable for 3 days at 4°C (Figure 3), sufficient to undertake non-crystallographic structural studies.

### Crystallization of the N-Terminal TDP-43 Dimerization Domain

**Figure 4:**
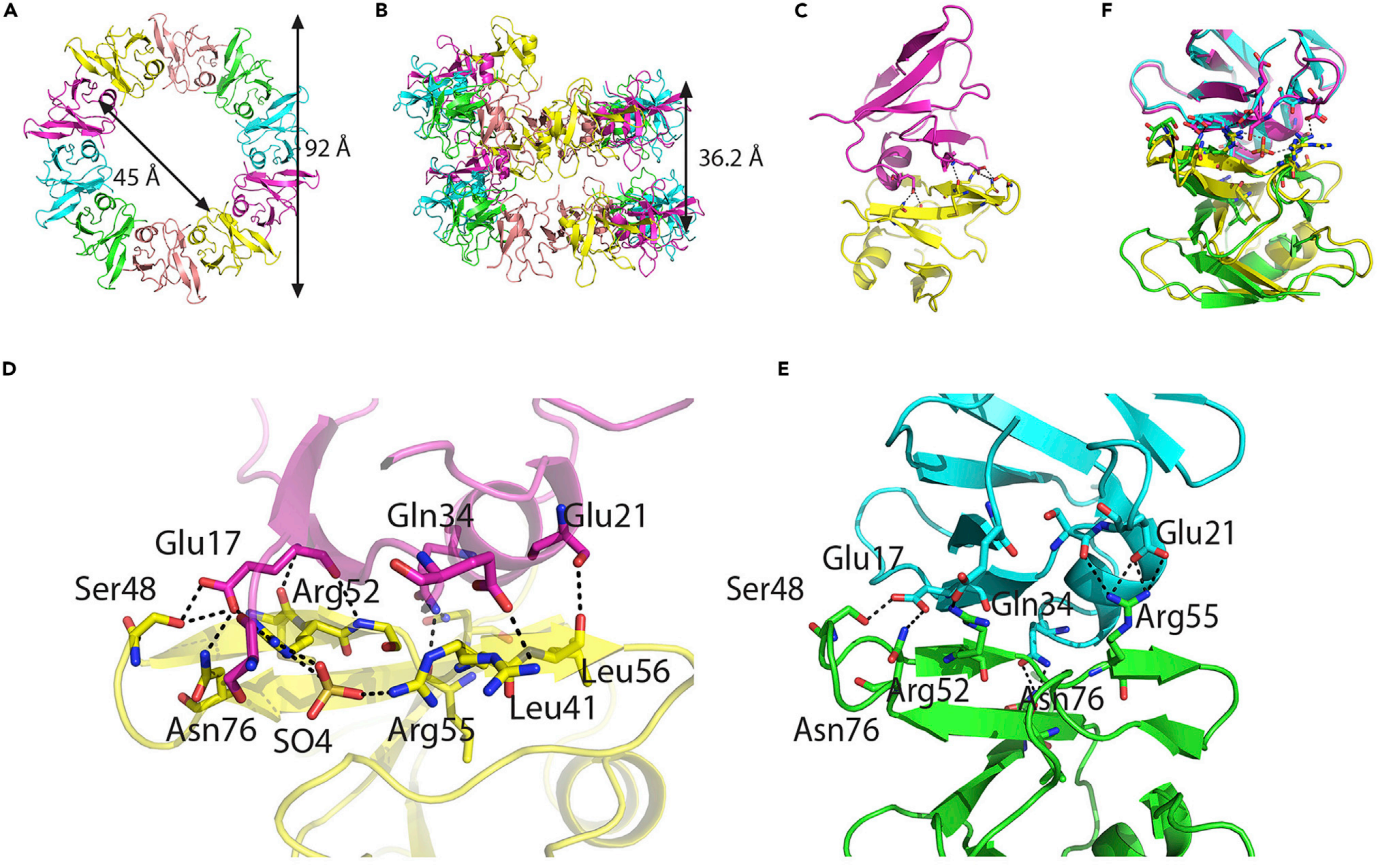
Crystal Structure of TDP-43 NTD at 2.55 Å Resolution Molecules of TDP-43 NTD are arranged in the crystal as parallel spirals. (A) Filaments from neighboring molecules arranged in head-to-tail fashion. Atoms of 10 molecules comprising two neighboring asymmetric units make one full turn of the spiral and are shown in different colors. The outside radius of the spiral is ~92 Å, whereas internal radius is 45 Å. (B) Side view of the spiral, showing two full turns with distance between two turns of the spiral 36.2 Å. (C) Cartoon representation of two neighboring TDP-43 NTD molecules. (D) Expanded view of the interface between molecules shown in (C). Amino acid side chains making intermolecular contacts are shown in stick representation in the corresponding domain color and labeled. Intermolecular hydrogen bonds are shown as dotted black lines. (E) The dimer interface described by Afroz et al. (2017) (PDB: 5MDI) and labeled as in (C). (F) Superposition between dimer interfaces shown in (D) and (E). Structures were aligned to one molecule rather than the whole dimer.

To elucidate the molecular basis of TDP-43 pathogenesis, a stable N-terminal 1-270 fragment was used for crystallization and formed crystals when grown at 19°C. The structure provided a surprise as only the NTD (1-80) was visible. Analysis of several crystallization drops by SDS-PAGE indicated the 30-kDa original protein to have shifted to approximately 23 kDa, which would indicate cleavage of RRM2. During 1 week of crystallization the protein appears to have auto-cleaved. The TDP-43 NTD crystallized in space group P2_1_2_1_2_1_ with five identical molecules in the asymmetric unit. The structure was solved by molecular replacement and refined to 2.55 Å resolution (Table 1). The NTD adopts a similar conformation as reported in a recent crystallographic structure obtained in P6_3_ space group (Afroz et al., 2017). A compact five-stranded β-sheet fold was observed with a two-turn alpha helix (Figures 4A–4C), and the negatively charged tail of each domain is packed against the positively charged C-terminal part (head-to-tail interaction).

**Table 1:** Crystallographic Data Collection and Refinement Statistics

	TDP43
**Data Collection**	

Space group	P2_1_ 2_1_ 2_1_

**Cell dimensions**	

a, b, c (Å)	34.637, 95.224, 157.558
α, β, γ (°)	90.00, 90.00, 90.00
Resolution (Å)^a^	78.90–2.55 (2.62–2.55)
R_merge_^a^	15.6 (0.986)
I/σI^a^	5.7(1.6)
CC_1/2_ (%)^a^	0.985(0.549)
Completeness (%)^a^	98.7(99.1)
Redundancy^a^	3.9(4.0)
Wilson B (Å^2^)	38.3

**Refinement**	

No. reflections	16,549
R_work_/R_free_	21.46/25.86

**No. of atoms**	

Protein	3,040
Ligand/ion	25
Water	235

**B-factors**	

Protein	50.78
Ligand/ion	94.12
Water	43.33

**RMS deviations**	

Bond lengths (Å)	0.0040
Bond angles (°)	1.256
PDB	6T4B

a values in paranthesis are for the outer shell of data

The head-to-tail interfaces between the molecules differ from those reported previously (Afroz et al., 2017). A sulfate ion binds at the interface between the molecules and is bonded to Arg52 and Arg55, which serves as a wedge, increasing the distance between the monomers. This binding of a sulfate ion to Arg52 and Arg55 (Figure 4D) rearranges their side chains preventing strong bonding between Arg52-Glu3 and Arg55-Glu21 reported in an earlier NTD structure (Afroz et al., 2017). These change the position of the N terminus toward the interface (Figures 4D and 4E), moving the Glu3 side chain away from Arg52 and making strong salt bonding with Arg52 impossible. The changes in the position of the monomers against each other alter the superhelical bundle arrangement observed previously (Afroz et al., 2017) (Figure S1). In our structure, bundles of the tight spirals are packed against six neighboring spirals (Figure S1C). Each spiral, with radius of 46 Å, has 10 NTD molecules in a full turn (Figures 4A, 4B, S1A, and S1B) with distance between two rings being ~36 Å. Analysis of the structural changes on sulfate binding confirms that ligand binding at the molecular interface could change the nature of the helical arrangements. These head-to-tail interactions that lead to parallel spirals are consistent with the view that physiological TDP-43 oligomerization is mediated by its NTD and may be key to prevent the formation of pathologic aggregates.

### Structural Characterization of Full-Length Tryptophan-Free TDP-43

**Figure 5:**
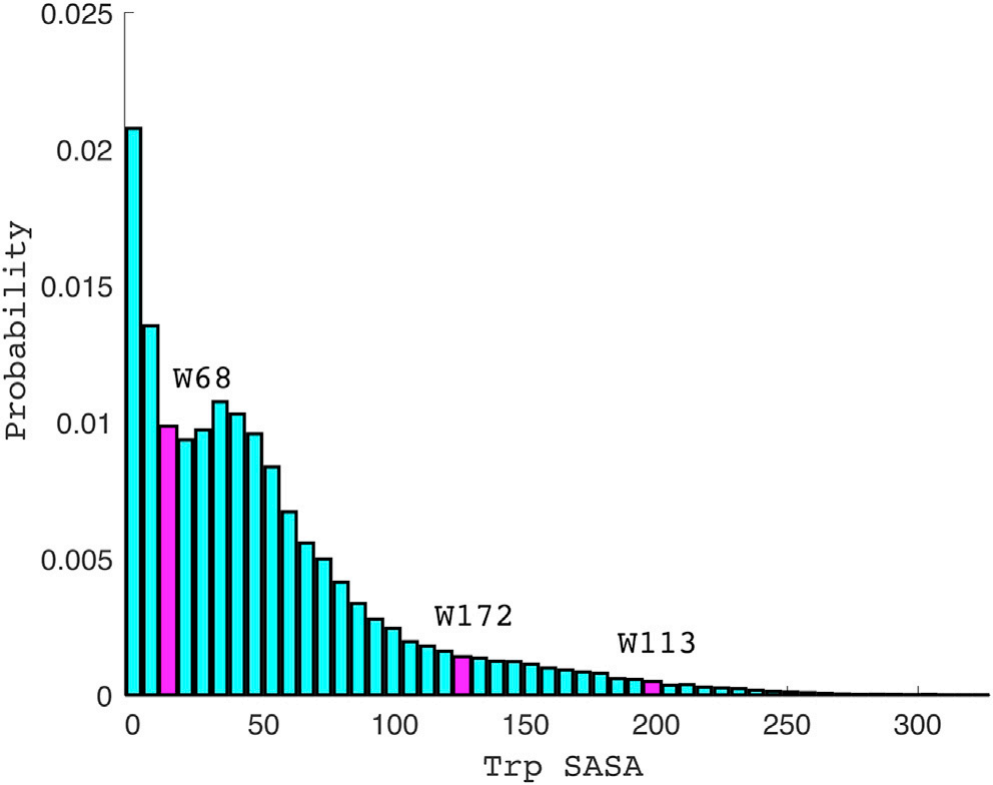
Distribution of Solvent Accessible Surface Area (SASA) of Tryptophans in a Non-redundant Database of Protein Structures SASA for three tryptophan residues found in structured domains of TDP43 is shown in magenta: W68 in the NTD and W113 and W172 in RRM1. Their cumulative percentile of exposure is 23.6%, 98.5%, and 91.8% respectively.

Solvent-accessible tryptophan residues within intrinsically disordered regions are unlikely to contribute to individual domain structures. It is, however, important to understand the perturbations caused by mutation of tryptophans within the NTD and RRM1 domain. Available RRM1 structures (Kuo et al., 2014, Lukavsky et al., 2013) show Trp178 has a role in nucleic acid binding but in the apo form both Trp113 and Trp172 side-chains protrude into solvent. Comparing tryptophan solvent accessible surface area in various proteins taken from a non-redundant database of 27,015 protein structures (He et al., 2014) indicates the RRM1 tryptophans to be unusually exposed (Figure 5). Thus, mutation of these residues is unlikely to change the overall RRM1 structure. The side chain of Trp68, found in NTD loop 5 adjacent to the domain core, forms hydrophobic interactions with other residues within the core (Figure S2) (Mompeán et al., 2016) and may support a homodimer interface (Afroz et al., 2017). Indeed, reducing homodimerization propensity may have aided our recovery of soluble and relatively stable full-length protein. Trp68 retains some solvent exposure (Figure 5), is surrounded by loop regions, whose mutation is thus unlikely to affect secondary structural elements that contribute to overall domain tertiary structure.

**Figure 6:**
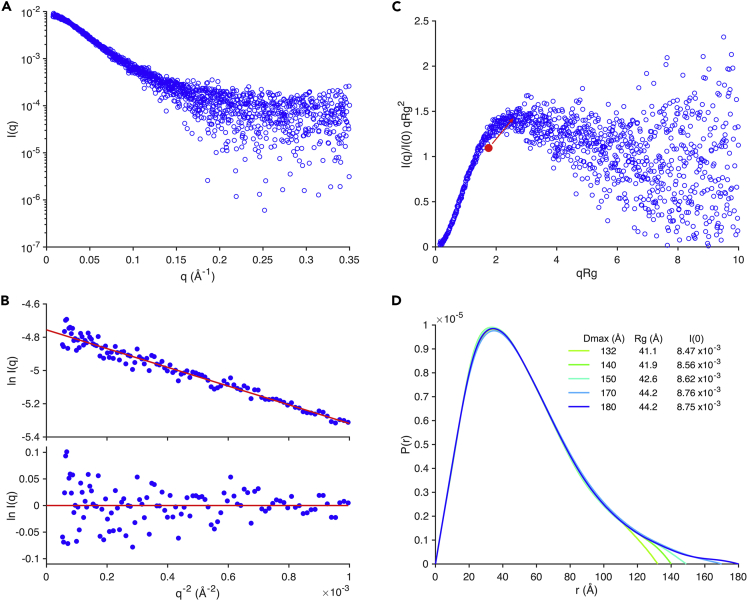
Full-Length TDP-43 X-Ray Scattering (A) Intensity plot of X-ray scattering by TDP-43_WtoA_. (B) TDP-43_WtoA_ Guinier plot (upper) and residuals (lower). R^2^ 0.974 over data range 0.3 < q.Rg < 1.29. Rg = 41.1 ± 0.4 Å (with error stated as SEM) as determined by m = -Rg^2^/3, where m is the gradient of the line, and I(0) = 8.60 × 10^−3^. Both plots indicate a monodisperse sample with little or no interparticle interference. (C) Guinier-based dimensionless Kratky plot showing that the peak maximum for TDP-43_WtoA_ is moved away from the point at √3 and 1.1 (highlighted in red) where globular proteins show a maximum. This is indicative of unstructured regions. The overall form of the curve, which does not return to the baseline after the initial peak but does not continue to increase with qRg, is also indicative of a protein with both folded and unfolded regions. (D) SAXS distance distribution functions (P(r)) for TDP-43_WtoA_. Multiple possible functions are plausible with a Dmax range 132–180 Å. P(r) functions with Dmax 132–150 Å have real space Rg and I(0) that correlate well with those from the Guinier approximation, whereas those with Dmax 170–180 Å show smooth transitions with the r scale. Dmax, Rg, and I(0) determined from each P(r) function are stated in the legend.

Using the expression protocol described above, and elaborated upon in the Transparent Methods section, we were able to produce sufficient detergent-free recombinant TDP-43_WtoA_ to perform chromatographic SAXS experiments. Size exclusion chromatograms of full-length TDP-43 (Figure S3) are slightly asymmetric. This could be interpreted as a fast oligomeric equilibrium, between monomer and dimer, for example, or an interaction with column media, which delays elution of some molecules. Sampling data points in the first and last thirds of a single TDP-43_WtoA_ elution indicates a decrease in mean radius of gyration (Rg) from 41.8 ± 3.5 Å to 38.9 ± 4.0 Å (Figure S3). Owing to this small change in Rg, and lack of distinct sub-populations within each profile, we averaged the data across two full TDP-43_WtoA_ elutions. This yielded good-quality data over an angular range of 0.0084–0.35 Å^−1^ (Figure 6A). Inspection of the Guinier plot (Figure 6B) indicates that TDP-43_WtoA_ is not aggregated and has Rg 41.1 Å. Rendering the experimental data as a dimensionless Kratky plot shows TDP-43_WtoA_ to have a high degree of unfolding but to not be completely disordered (Figure 6C). This is expected given the presence of previously characterized modular nucleic acid binding and dimerization domains (Afroz et al., 2017, Austin et al., 2014, Lukavsky et al., 2013). Assignment of largest intramolecular distances (Dmax) is difficult for disordered proteins, and this is the case for TDP-43_WtoA_, with possible Dmax values ranging from 132 to 180 Å (Figure 6D).

**Figure 7:**
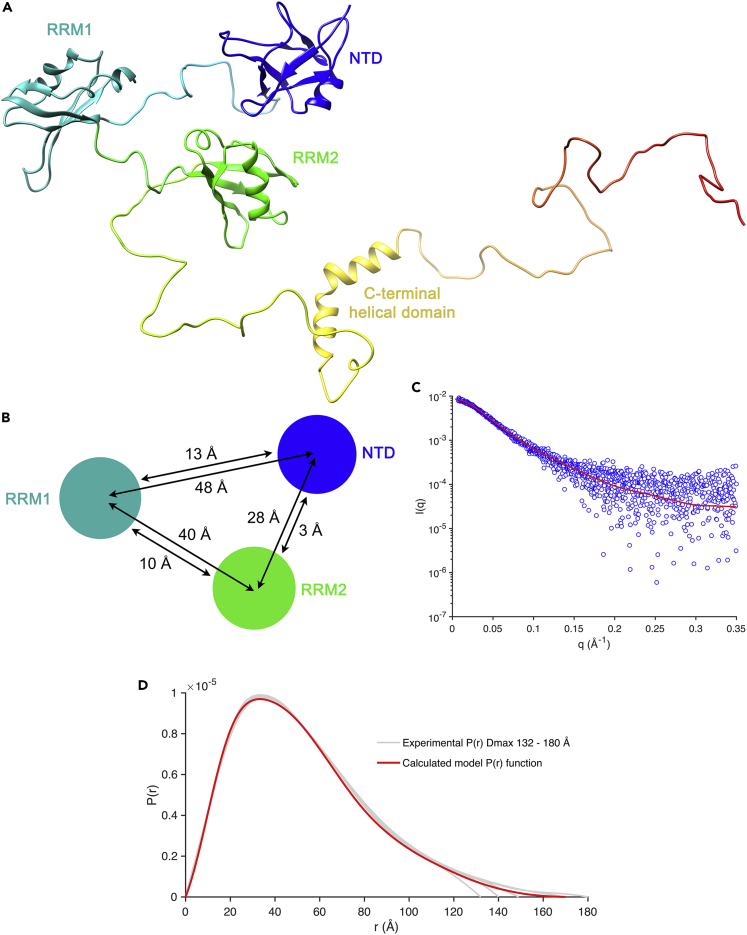
Model of Full-Length TDP-43 Refined Against SAXS Data (A) Model of full-length TDP-43_WtoA_. Model has Rg 41.9 Å and Dmax 154 Å. (B) Schematic of the above showing center of mass and closest contact intradomain distances. (C) Model fit to experimental intensity scattering data χ^2^ 1.10. (D) Distance distribution functions for experimental data showing solutions with variable possible Dmax compared with that for the model.

Using available structures for RRMs, dimerization, and helical domains in conjunction with linker peptides synthesized *in silico* we generated initial models of full-length TDP-43_WtoA_ (Figure S4) and refined them against our experimental SAXS data to yield a conformationally optimized model (Figure 7A). The monomeric model generated has little contact between domains except between the NTD and RRM2 (Figure 7B). Calculation of its scattering profile indicates a very good fit to the experimental data χ^2^ 1.1 (Figure 7C). The experimentally determined Rg agrees with the model within 0.8 Å (41.1 and 41.9 Å, respectively). Comparison of the distance distribution functions of our monomeric, full-length TDP-43_WtoA_ model with that derived from experimental data also indicates exceptionally good correlation (Figure 7D). Models of TDP-43_WtoA_ where dimerization was enforced through the NTD had consistently poor fit to the experimental data, χ^2^ of 1.5 and Rg 49.7 Å.

## Discussion

Although the structures of individual domains of TDP-43 have been elucidated using several techniques that have revealed some aspects of the molecular functions of this protein (Afroz et al., 2017, Chiang et al., 2016, Guenther et al., 2018, Kuo et al., 2014, Mompeán et al., 2016), the structure of full-length TDP-43 has been refractory to characterization due to the difficulty of purifying soluble and stable protein in sufficient amounts for analysis (Johnson et al., 2009, Kitamura et al., 2018, Li et al., 2017). A recent report overcame this challenge using denaturing conditions, but this could change the native TDP-43 structure (Vivoli Vega et al., 2019). In this work, we describe full-length wild-type TDP-43 and TDP-43_WtoA_, both successfully purified by non-denaturing methods. SAXS data of TDP-43_WtoA_ reveal the conformation of the full-length protein in solution. The region of TDP-43 comprising the NTD, RRM1, and RRM2 adopts a compact triangular structure, whereas the position of the C terminus is variable.

A fragment of TDP-43 comprising residues 1-270 including NTD and RRMs was used in crystallization experiments and the structure of the NTD (residues 1-80) was elucidated at 2.55 Å resolution. The protein appears to have been auto-cleaved at N terminal to RRM2 during the 7 days required for crystallization to produce diffraction-quality crystals, whereas RRM1 is conformationally mobile *in crystallo* and therefore not visible. The TDP43 NTD has been reported to be essential for dynamic TDP-43 oligomerization that may prevent the aggregation-prone C terminal from forming pathogenic and irretrievable TDP-43 aggregates. This crystal structure shows that the superhelical format arises from head-to-tail interactions between NTD molecules. The SAXS model implies that the peptide chain linking NTD and RRM1 domains is longer than other domains, so it is likely to be disordered when forming higher-order structures.

The ability to obtain full-length TDP-43 in a stable form without denaturing conditions opens possibilities for extensive biophysical studies on both the wild-type and C-terminal mutants, which are known to exert greater aggregation propensity (Cao et al., 2019). We propose that the approaches used here may have general applicability and may prove useful for other aggregation-prone proteins where a significant proportion of the macromolecule is classified as “unstructured,” i.e., having a lower folding probability. Enabling the purification of stable complexes via mutation of residues that cause precipitation or aggregation has recently proven key to our description of the functional complexes between SOD1 and its cognate chaperone (Sala et al., 2019). Similarly, use of detergents for soluble proteins should enable high-concentration structural studies for such systems, providing an essential platform for molecular understanding of pathogenic properties and possible therapeutic solutions.

### Limitations of the Study

**Figure 8:**
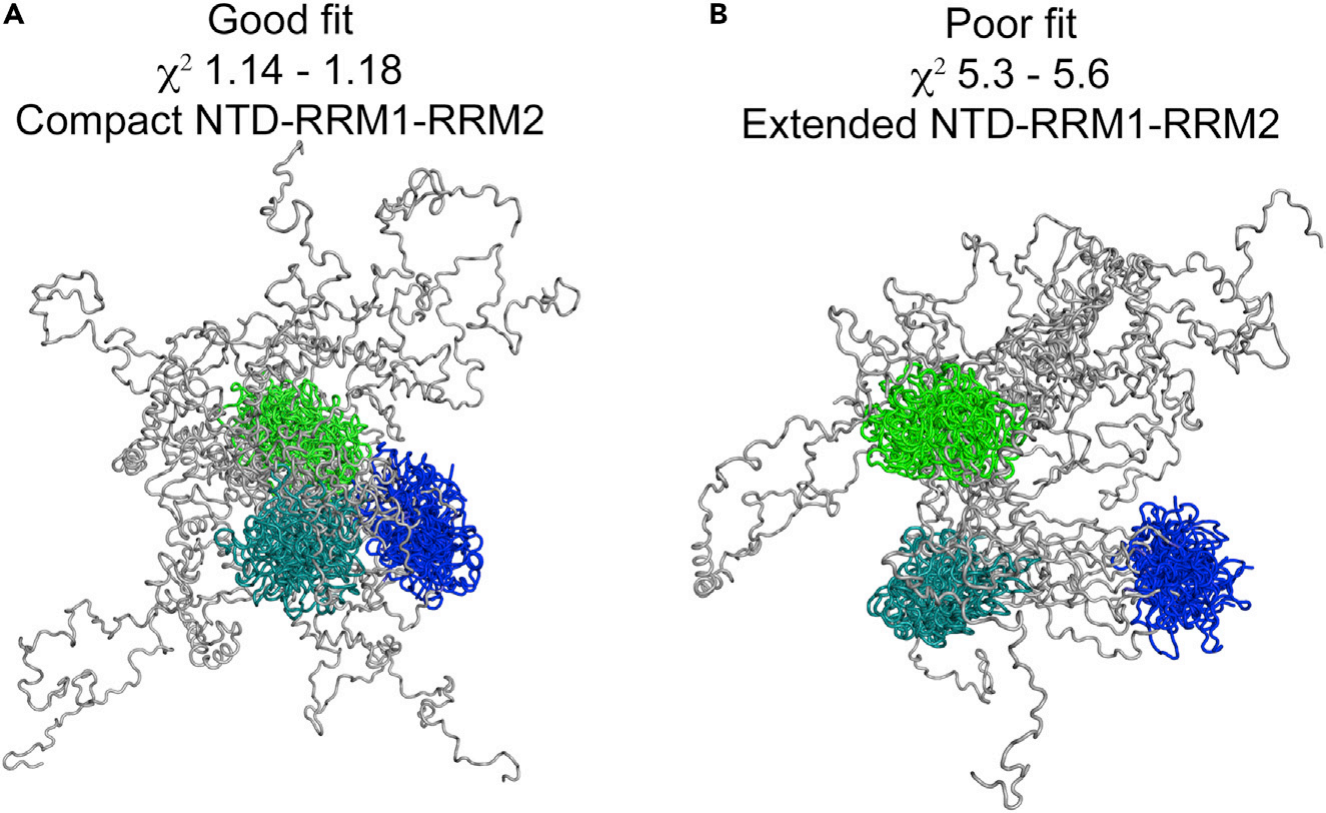
Comparison of Full-Length TDP-43_WtoA_ Models with Both Good and Poor Fit to Experimental SAXS Data (A) Ten TDP-43_WtoA_ models with highest goodness of fit to the experimental data from a pool of 7,000 aligned to amino acids 1-258 of the model presented in Figure 7A. (B) Ten models with lowest goodness-of-fit to experimental data aligned to amino acids 1-258 of the model with highest χ^2^.

A population of intrinsically disordered sequences are, by their nature, very unlikely to simultaneously occupy the same conformation. Although the model presented in Figure 7A is our best representation of the TDP-43 structure, it is clearly a snapshot of a very dynamic system. The model presented in Figure 7A has an unrestrained and predominantly disordered C-terminal region but, as noted, a relatively compact N-terminal region comprising the NTD, RRM1, and RRM2. Disordered parts of the protein contribute less to scattering intensity than globular domains and are poorly defined by scattering data. To determine how variable the spatial arrangement presented in Figure 7A may be, we performed long molecular dynamics simulations using our optimized model as a starting structure. Figure 8 shows that positioning of the disordered C terminus has little effect on the fit to experimental data. However, those structures that fit the data poorly exhibit an increase in the Rg value for the whole molecule and the Rg representing NTD, RRM1, and RRM2 domains (amino acids 1-258) (Figures S5A and S5B and Table S1). For these models, inter-domain linkers are also found in an extended conformation (Figures S5C–S5E and Table S1) and separation between domains is therefore maximized. Conversely, for models that fit the data well, the globular domains occupy a compact conformation, which matches that presented in Figure 7A (Figures S5A and S5B and Table S1). For both well-fitting and poorly fitting model groups the specific orientation of globular domains cannot be accurately defined but the geometry of domain positions can.

### Resource Availability

#### Lead Contact

Further information and requests for resources and reagents should be directed to and will be fulfilled by the Lead Contact, S. Samar Hasnain (s.s.hasnain@liverpool.ac.uk).

#### Materials Availability

All unique and stable reagents generated in this study are available from the Lead Contact without restriction.

#### Data and Code Availability

The atomic coordinate and structure factor of the NTD (NTD) of TDP-43 have been deposited in the Protein DataBank (http://www.rcsb.org/) under the accession code 6T4B.

Experimental SAXS data and TDP-43 models are available from the corresponding author on request.

## Methods

All methods can be found in the accompanying Transparent Methods supplemental file.

## Supplemental Information

Document S1. Transparent Methods, Figures S1–S5, and Table S1
