# Supplementary material for: Purification and Structural Characterization of Aggregation-Prone Human TDP-43 Involved in Neurodegenerative Diseases
Source: iScience. 2020 May 15;23(6):101159. doi: 10.1016/j.isci.2020.101159 (PMC7262455; doi:10.1016/j.isci.2020.101159)
Supplement: Document S1. Transparent Methods, Figures S1–S5, and Table S1 [file mmc1.pdf]

iScience, Volume *23*

Supplemental Information

Purification and Structural Characterization of Aggregation-Prone Human TDP-43 Involved in Neurodegenerative Diseases

Gareth S.A. Wright, Tatiana F. Watanabe, Kangsa Amporndanai, Steven S. Plotkin, Neil R. Cashman, Svetlana V. Antonyuk, and S. Samar Hasnain

Supplemental Information

Supplemental Figures

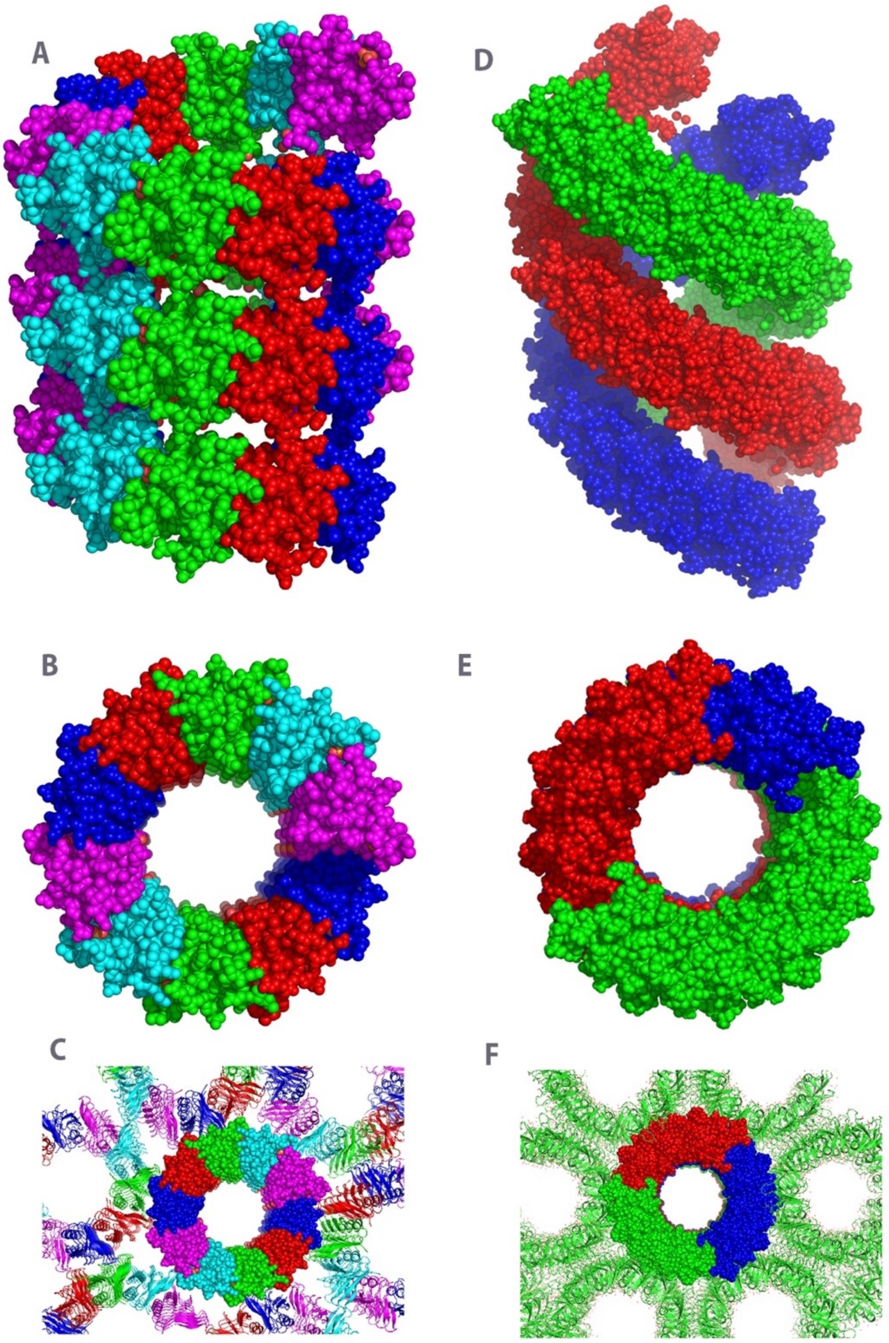

Figure S1. Arrangement of TDP-43 NTD domains within different crystal forms. Continuous spiral with 10 NDTs in one turn of the spiral A) side view of the spiral and, B) top view. C) Packing of the crystal from the same direction as B. The identical chains are illustrated in the same colour. D) Super-helical arrangement (PDB: 5MDI) from the side and, E) the top. F) Packing of the crystals containing filaments. Three different representative filaments containing 10 domains are coloured in red, green and blue.

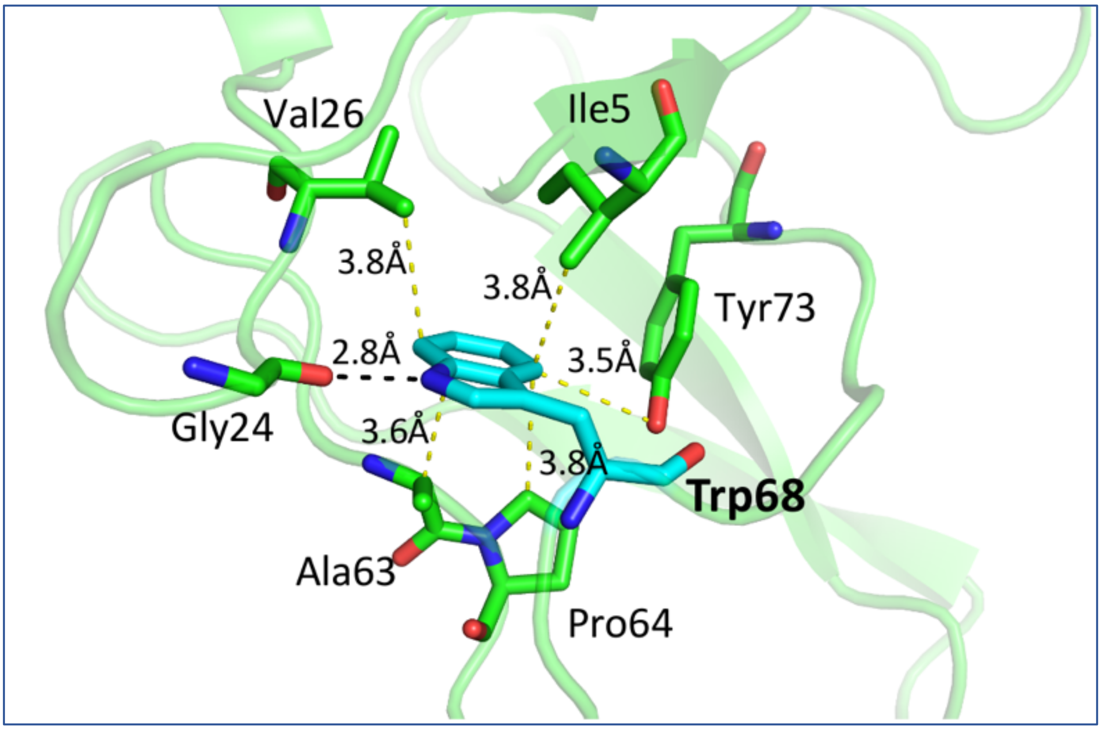

Figure S2. Hydrogen bond formation and hydrophobic interactions formed by TDP-43 N-terminal domain Trp68. Trp68 and surrounding residues are shown in cyan and green, respectively. Hydrogen bond and hydrophobic contacts are illustrated as black and yellow dashed lines, respectively.

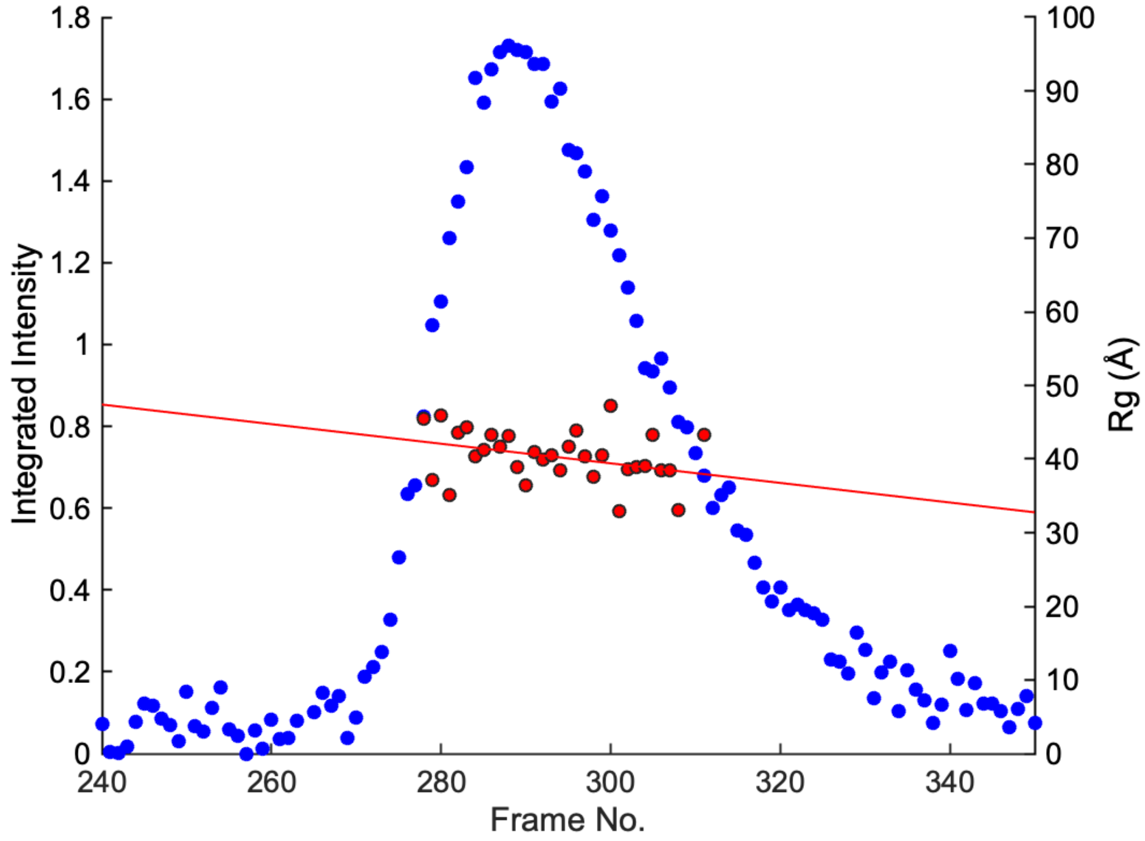

Fig S3. Chromatographic SAXS parameters for TDP-43$_{WtoA}$. Integrated intensity is plotted in blue. Radius of gyration is plotted in red. The slope of the Rg plot is shown as a red line which has gradient coefficient -0.13 and R^2 0.12. Data points in the first third of the elution have Rg 41.8 ± 3.5 Å while those in the last third have Rg 38.9 ± 4.0 Å, with error quoted as standard deviation.

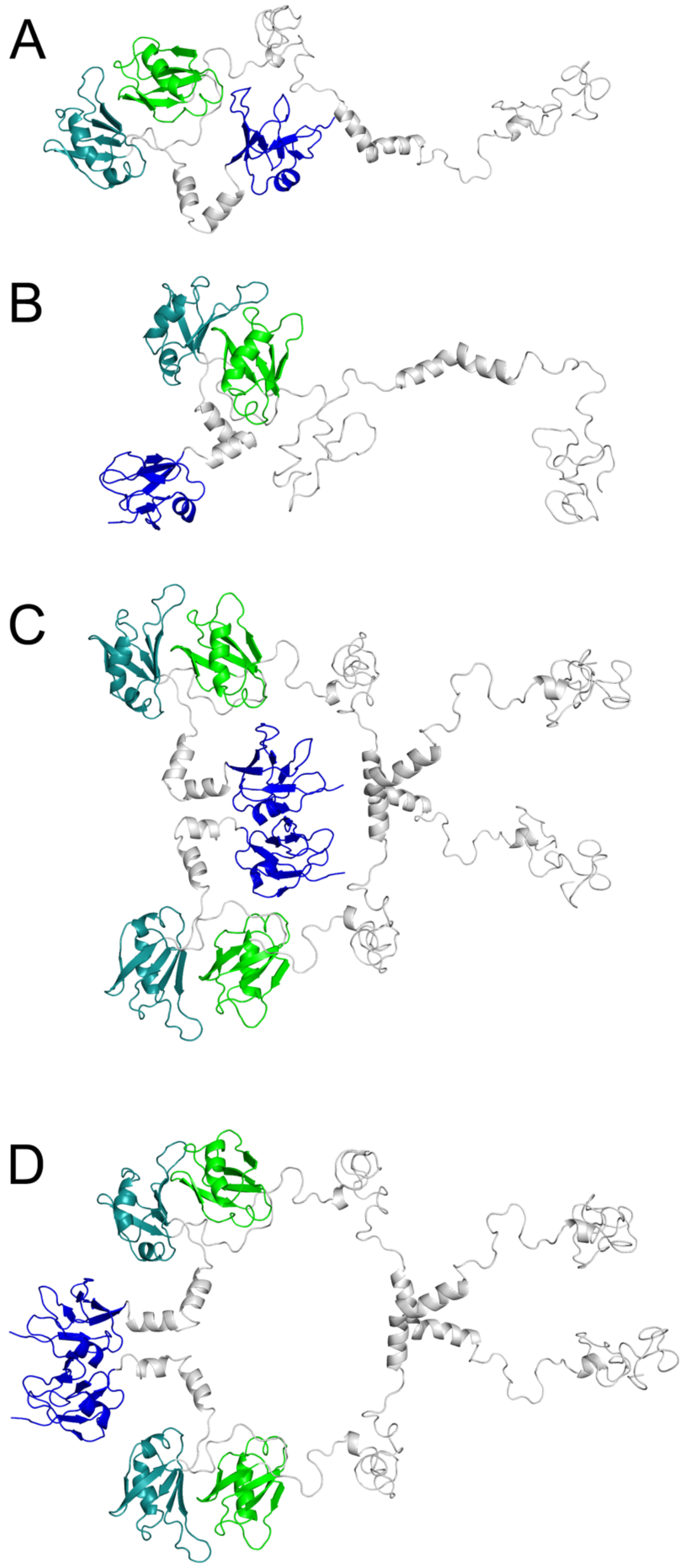

Figure S4. Comparison of initial models of full-length TDP-43. A and B) Monomeric TDP-43. C and D) Dimeric TDP-43.

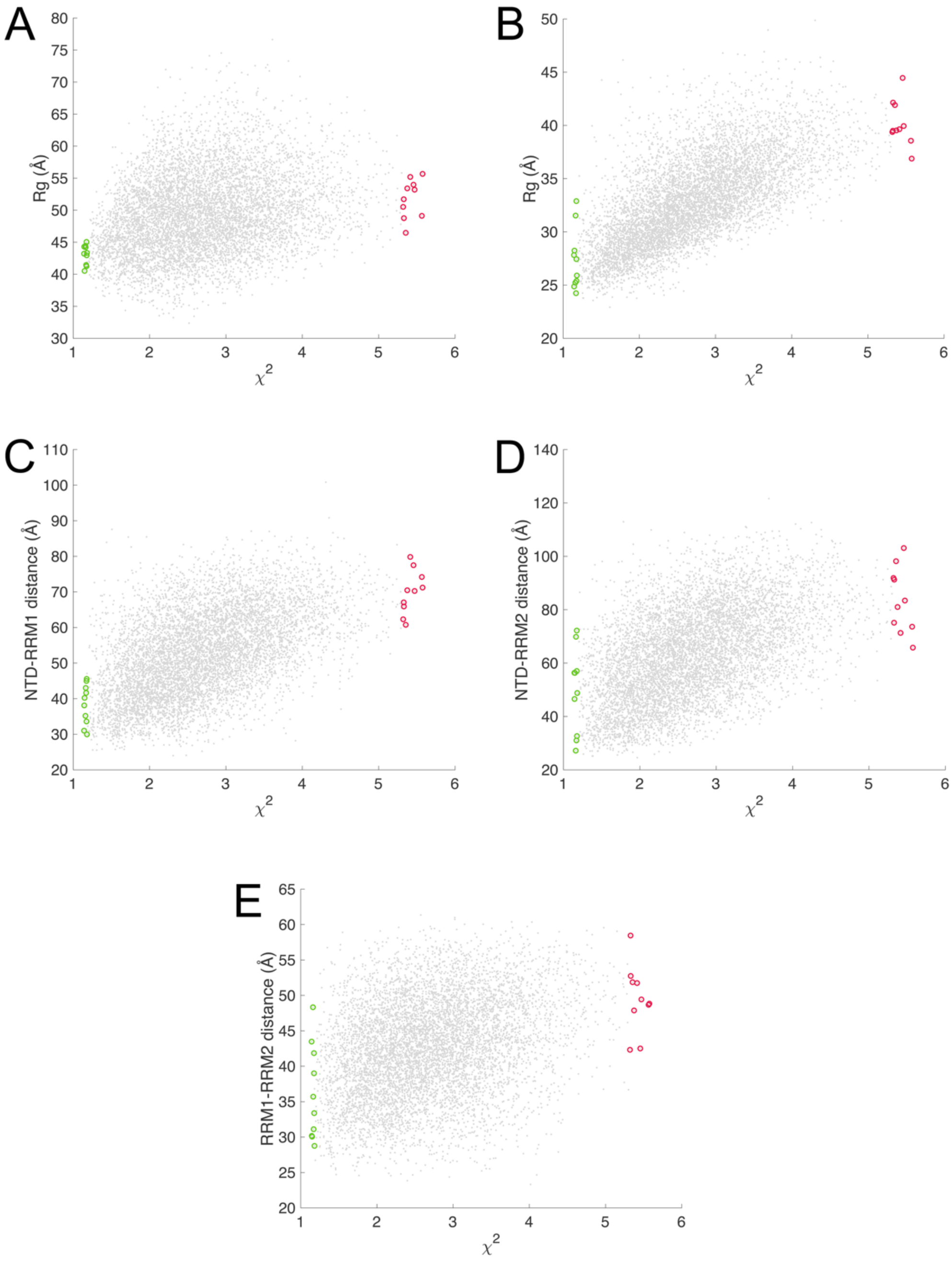

Figure S5. Analysis of goodness-off fit to experimental SAXS data versus size parameters for 7000 TDP-43$_{WtoA}$ models. A) The relationship between full-length TDP-43$_{WtoA}$ model Rg to χ^2. B) The relationship between Rg of the N-terminal region of TDP-43$_{WtoA}$ molecule comprising amino acids 1-258 (NTD-RRM1-RRM2) to χ^2. C) The relationship between TDP-43$_{WtoA}$ NTD-RRM1 distance and χ^2. D) The relationship between the TDP-43$_{WtoA}$ NTD-RRM2 distance and χ^2. E) The relationship between the TDP-43$_{WtoA}$ RRM1-RRM2 distance and χ^2. Parameters for structures presented in Figure 8A and 8B are highlighted with green and red respectively with numerical data given in Table S1.

	10 well-fitting models	10 poorly fitting models	Optimised model (Figure 7A)
χ^2	1.16 ± 0.01	5.41 ± 0.10	1.10
**Rg of full-length TDP43 (Å)**	43.1 ± 1.5	51.8 ± 3.0	41.9
**Rg of amino acids 1-258 (Å)**	27.4 ± 2.9	40.2 ± 2.1	25.4
**NTD-RRM1 distance (Å)**	38.3 ± 5.7	70.0 ± 6.2	48.2
**NTD-RRM2 distance (Å)**	49.8 ± 15.7	83.5 ± 12.3	28.2
**RRM1-RRM2 distance (Å)**	36.2 ± 6.7	49.4 ± 4.7	40.3

Table S1. Comparison of size parameters for the models presented in Figures 7 and 8. Error measurements are given as standard deviation.

Transparent Methods

Expression and purification

Full-length wild-type TDP-43 (wtTDP-43) and TDP-43 construct containing W67A, W113A, W172A, W334A, W385A and W412A mutations (TDP-43_{WtoA}) were synthesised *de novo* with an N-terminal hexa-his tag and tobacco etch virus (TEV) cleavage site in pET-28a(+) vector. This expression plasmid was transformed into BL21 (DE3) *E. coli* and pre-cultured at 37 °C overnight in LB media with 50 μg/ml of kanamycin. 25 ml of this pre-culture was added into 1 litre of LB media and the culture was incubated at 37 ºC in a shaker until the optical density at 600nm was 0.7. The culture was cooled for 30 min at 4 °C. IPTG was added to a final concentration of 0.5 mM to induce protein expression and the flasks were kept in shaker overnight at 18 ºC. The cells were then harvested by centrifugation.

1 g of cells was resuspended in 5 mL water and incubated at 4 °C for 30 minutes before sonication on ice. The cell lysate was clarified by centrifugation at 35,000 g for 1 hour before adding 50mM sodium phosphate pH 8.0 and 0.2% sarkosyl. A nickel-NTA IMAC column was equilibrated with 50 mM sodium phosphate pH 8.0, 300 mM sodium chloride, 5 mM imidazole, 0.2% sarkosyl prior to application of the soluble fraction at room temperature. Protein was eluted from the column with 150-500 mM imidazole in 50 mM sodium phosphate pH 8.0, 300 mM sodium chloride, 5 mM DTT and dialysed overnight at 4 °C with the addition of TEV protease against the same buffer without imidazole. The cleaved protein was then filtered through the NiNTA column again with wtTDP-43 now passing directly through. It was then concentrated and applied to a Superdex 75 16x600 mm size exclusion chromatography column with 0.5 ml/min flow of 20 mM Tris-HCl pH 8.0, 300 mM NaCl, 5 mM DTT, 0.2% sarkosyl. Fractions were analysed by SDS-PAGE and those found to contain full-length wtTDP-43 were pooled, concentrated and stored at 4 °C.

Full-length TDP-43_{WtoA} and truncated amino acids 1-270 wild-type TDP-43 were expressed and purified as above protocol without using sarkosyl, but cell lysis was carried out in 50 mM sodium phosphate pH 8.0, 300 mM sodium chloride, 5 mM imidazole, 5 mM DTT, complete protease inhibitor cocktail (Roche), 1 mM PMSF, 50 ug/ml lysozyme instead of water.

Crystallisation

A 1-270 amino acid fragment of TDP-43 protein at 40 mg/ml concentration was crystallised at 19 °C by the hanging drop method. 1 μL of protein was mixed with 1 μL of ready-made solution

F2 from PACT premier screen (Molecular Dimentions) and equilibrated over the reservoir solution, containing 0.2M Sodium bromide, 0.1M Bis-Tris propane 6.5, 20% PEG 3350. Crystals were flash frozen in liquid nitrogen using reservoir solution with additional 10% glycerol.

Data collection, structure determination and refinement

Data were collected at DIAMOND synchrotron, beamline I03 using x-rays of 0.9763 Å wavelength with PILATUS 6M detector to 2.55 Å resolution. Data were integrated with iMosflm (Battye et al., 2011) and scaled with Aimless (Evans, 2011) software as part of the CCP4 package. The structure was solved by Molrep software (Vagin and Teplyakov, 2010) with starting model TDP-43 structure (PDB:5MDI chain A) and refined using Refmac5 (Murshudov et al., 2011) with applied NCS symmetry. TLS refinement was implemented towards the end of the refinement. Data collection and refinement statistics are presented in Table 1.

Small-angle x-ray scattering data collection

Chromatographic SAXS data for full-length TDP-43_{WtoA} was collected at Diamond Light Source on beamline B21. Data was acquired following a chromatographic step where 45 μl of TDP-43_{WtoA} at 5 mg/ml was loaded onto a Superdex 200 10x300 mm size exclusion chromatography column at room temperature. The protein was eluted at a flow rate of 200 μl/min and directly exposed to x-rays. Blank frames were taken prior to protein elution for buffer subtraction purposes. Individual frames recorded over a single chromatography run were averaged based on Rg values and overall similarity in a correlation map. Two separate chromatography runs were performed and the results of each were averaged to give the final scattering profile. Averaging was performed with ScÅtter. The scattering curve of full-length TDP-43 was observed in ScÅtter, Primus (Konarev et al., 2003) and Matlab where the Guiner approximation was performed and Rg determined. GNOM (Svergun, 1992) was used to determine distance distribution functions.

Structure modelling and refinement against SAXS data

An initial model of full-length TDP-43 was constructed from NMR structures of the NTD (Mompeán et al., 2016) (2N4P); RRM1 and RRM2 (Lukavsky et al., 2013) (4BS2); and an helical structure formed by residues 321-343 in the C-terminal tail (Jiang et al., 2016) (2N3X) shown to be partially populated (Conicella et al., 2016). Pepfold (Maupetit et al., 2009) was used to generate the remaining linkers between these structured segments and Modloop

(Fiser and Sali, 2003) was used to link each component to form a continuous polypeptide. Tryptophan residues were mutated to alanine using Coot (Emsley and Cowtan, 2004).

Two different starting models were refined against experimental SAXS data using CNS (Brunger, 2007), as described previously (Wright et al., 2016, 2018). Domains defined above were initially treated as free-floating rigid bodies and inter-domain linkers allowed to move freely over 750 ps simulations at 300,000 K. Over the course of the simulation 1000 structures were written out and compared with the experimental scattering data with FoXS (Schneidman-Duhovny et al., 2016). This global refinement of domain positions was followed by fine-tuning of the position and orientation of individual domains. Here, the majority of the protein was fixed in space while one domain and its surrounding linkers were allowed to move. Each step in this process was performed independently with different initial trajectories 6 to 8 times. The structure that fit the data best was taken forward for further optimisation. To ensure that conformational space had been adequately sampled during this process, a 3.75 ns simulation was performed starting from our optimised model with all domains able to move freely. This was independently repeated with different seed trajectories 7 times. Domain centres of mass were calculated using CNS.

Experimental SAXS data and derived models are available upon request.

Solvent accessible surface area calculations

Apo structures (protein only without nucleic acid or other ligand) are used to calculate the SASA of tryptophans in a non-redundant database of 27,015 structures taken from the Protein Data Bank using MUFOLD-DB (He et al., 2014), with 70% sequence identity. The SASA of amino acids was calculated using tcl scripts in VMD (Humphrey et al., 1996) and confirmed using built-in commands in GROMACS modified with improved resolution of sampling points. Hydrogens are added using GROMACS before the SASA calculation.

Supplemental References

Battye, T.G.G., Kontogiannis, L., Johnson, O., Powell, H.R., and Leslie, A.G.W. (2011). iMOSFLM: A new graphical interface for diffraction-image processing with MOSFLM. Acta Crystallographica Section D: Biological Crystallography *67*, 271–281.

Brunger, A.T. (2007). Version 1.2 of the Crystallography and NMR system. Nat Protoc *2*, 2728–2733.

Conicella, A.E., Zerze, G.H., Mittal, J., and Fawzi, N.L. (2016). ALS Mutations Disrupt Phase Separation Mediated by α-Helical Structure in the TDP-43 Low-Complexity C-Terminal Domain. Structure *24*, 1537–1549.

Emsley, P., and Cowtan, K. (2004). Coot: Model-building tools for molecular graphics. Acta Crystallographica Section D: Biological Crystallography *60*, 2126–2132.

Evans, P.R. (2011). An introduction to data reduction: Space-group determination, scaling and intensity statistics. Acta Crystallographica Section D: Biological Crystallography *67*, 282–292.

Fiser, A., and Sali, A. (2003). ModLoop: automated modeling of loops in protein structures. Bioinformatics *19*, 2500–2501.

Humphrey, W., Dalke, A., and Schulten, K. (1996). VMD: visual molecular dynamics. J Mol Graph *14*, 33–38, 27–28.

Jiang, L.-L., Zhao, J., Yin, X.-F., He, W.-T., Yang, H., Che, M.-X., and Hu, H.-Y. (2016). Two mutations G335D and Q343R within the amyloidogenic core region of TDP-43 influence its aggregation and inclusion formation. Sci Rep *6*, 1–11.

Konarev, P.V., Volkov, V.V., Sokolova, A.V., Koch, M.H.J., and Svergun, D.I. (2003). PRIMUS: a Windows PC-based system for small-angle scattering data analysis. J. Appl. Cryst. *36*, 1277–1282.

Maupetit, J., Derreumaux, P., and Tuffery, P. (2009). PEP-FOLD: an online resource for de novo peptide structure prediction. Nucleic Acids Res. *37*, W498-503.

Murshudov, G.N., Skubák, P., Lebedev, A.A., Pannu, N.S., Steiner, R.A., Nicholls, R.A., Winn, M.D., Long, F., and Vagin, A.A. (2011). REFMAC5 for the refinement of macromolecular crystal structures. Acta Crystallographica Section D: Biological Crystallography *67*, 355–367.

Schneidman-Duhovny, D., Hammel, M., Tainer, J.A., and Sali, A. (2016). FoXS, FoXSDock and MultiFoXS: Single-state and multi-state structural modeling of proteins and their complexes based on SAXS profiles. Nucleic Acids Res. *44*, W424-429.

Svergun, D.I. (1992). Determination of the regularization parameter in indirect-transform methods using perceptual criteria. J. Appl. Cryst. *25*, 495–503.

Vagin, A., and Teplyakov, A. (2010). Molecular replacement with MOLREP. Acta Crystallographica Section D: Biological Crystallography *66*, 22–25.

Wright, G.S.A., Antonyuk, S.V., and Hasnain, S.S. (2016). A faulty interaction between SOD1 and hCCS in neurodegenerative disease. Sci Rep *6*, 27691.

Wright, G.S.A., Saeki, A., Hikima, T., Nishizono, Y., Hisano, T., Kamaya, M., Nukina, K., Nishitani, H., Nakamura, H., Yamamoto, M., et al. (2018). Architecture of the complete oxygen-sensing FixL-FixJ two-component signal transduction system. Sci. Signal. *11*, eaaq0825.
